# Suitability of the Cyclic Voltammetry Measurements and DPPH• Spectrophotometric Assay to Determine the Antioxidant Capacity of Food-Grade Oenological Tannins

**DOI:** 10.3390/molecules24162925

**Published:** 2019-08-13

**Authors:** Arianna Ricci, Giuseppina Paola Parpinello, Nemanja Teslić, Paul Andrew Kilmartin, Andrea Versari

**Affiliations:** 1 Department of Agricultural and Food Sciences, Alma Mater Studiorum—University of Bologna, Piazza Goidanich 60, 47521 Cesena, FC, Italy; 2 Institute of Food Technology, University of Novi Sad, Bulevar cara Lazara 1, 21000 Novi Sad, Serbia; 3 School of Chemical Sciences, The University of Auckland, Private Bag 92019, Auckland 1142, New Zealand

**Keywords:** oenological tannins, antioxidant activity, 2,2-diphenyl-1-picrylhydrazyl, cyclic voltammetry, glassy carbon electrode

## Abstract

Twenty commercially available oenological tannins (including hydrolysable and condensed) were assessed for their antiradical/reducing activity, comparing two analytical approaches: The 2,2-diphenyl-1-picrylhydrazyl (DPPH•) radical scavenging spectrophotometric assay and the cyclic voltammetry (CV) electrochemical method. Electrochemical measurements were performed over a −200 mV–500 mV scan range, and integrated anodic currents to 500 mV were used to build a calibration graph with (+)-catechin as a reference standard (linear range: From 0.0078 to 1 mM, R^2^ = 0.9887). The CV results were compared with the DPPH• assay (expressed as % of radical scavenged in time), showing high correlation due to the similarity of the chemical mechanisms underlying both methods involving polyphenolic compounds as reductants. Improved correlation was observed by increasing the incubation time with DPPH• to 24 h (R^2^ = 0.925), demonstrating that the spectrophotometric method requires a long-term incubation to complete the scavenging reaction when high-molecular weight tannins are involved; this constraint has been overcome by using instant CV measurements. We concluded that the CV represents a valid alternative to the DPPH• colorimetric assay, taking advantage of fast analysis and control on the experimental conditions and, because of these properties, it can assist the quality control along the supply chain.

## 1. Introduction

The word ‘tannins’ designates a class of polyphenolic compounds characterized by macromolecules and high-molecular weight polymers (500 Da–2000 Da) including complex structures with variable degrees of polymerization, arranged through condensed and hydrolysable chemical bonds. Tannins are synthesized by plants as a defense against pathogens; in fact, they are good electron-hydrogen donors and protect vegetable tissues by neutralizing oxidative molecules (mainly peroxides and reactive oxygen species, ROS) which are formed under biological stress conditions. Due to these properties, tannins are considered natural valuable compounds and they can be isolated from vegetable tissues using suitable extraction methods in their technological exploitation [1,2].

According to their chemical structures, tannins can be classified into two major groups, condensed tannins or proanthocyanidins (the latter name refers to their ability to release anthocyanidins under mild temperature and acidic conditions), and hydrolysable tannins, which are polyesters of gallic, ellagic, and hexahydroxydiphenoyl acids. When extracts are obtained from the raw vegetal sources, classification could be performed according to their botanical origin [3,4].

Tannins from grapefruit, tea leaves, oak wood, chestnut wood, gallnuts, and tara are traditionally exploited in the wine industry; they are available as lyophilized powders or stabilized solutions and directly added to wines and musts as ‘processing aids’ for the precipitation of excess proteinaceous matter. Regardless of the role of tannins in oenology, which is covered by current regulations (OIV International Code of Oenological Practices, 3.2 Clarification of Wine), these compounds play a key role on the chemical stability and sensory properties of wine. The use of commercial formulations is recommended for several purposes, including clarification and microbiological stabilization of musts, chemical and color stabilization of wines, strengthening of light-bodied wines [5]. Among these, the antioxidant activity constitutes a challenging opportunity for the technological exploitation of tannins in oenology, to assist or replace the use of sulphur dioxide for preventing oxidative damage in wine [6,7].

For a deeper insight on the reactivity of tannins we need to consider their composition and molecular structures, including the extent of the polymerization, substituents and steric hindrance, and the chemical reactivity involved.

**Figure 1:**
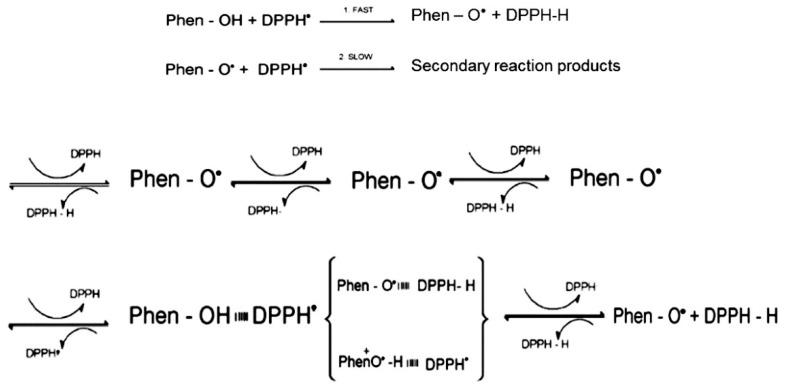
Schematic representation of the reaction between 2,2-diphenyl-1-picrylhydrazyl (DPPH)• radical and a generic polyphenolic compound (Phen-OH).

From a chemical perspective, the antioxidant activity of polyphenols occurs by the neutralization of ROS, which can be generated in wines and musts in the presence of catalysts and/or catalytic conditions (Figure 1), through hydrogen (HT) or electron transfer mechanism (ET). Both transfer mechanisms are dependent on the experimental conditions, i.e., the pH value of the solution or the polarity of the medium [8,9].

Targeted analytical assays have been developed to assess the antioxidant activity of polyphenolic compounds, providing a total antioxidant capacity (TAC) value in standard solutions and in real matrices. These assays are based on the ability of antioxidants to reduce Cu(II) and Fe(III) transition metals, responsible for the catalytic initiation of the oxidative chain, or to scavenge radicals, which are responsible for the oxidation of organic substrates. Among these, the 2,2-diphenyl-1-picrylhydrazyl (DPPH•) assay is based on the use of a stable synthetic radical in solution, with an unpaired valence electron at the nitrogen bridging atom. DPPH• is neutralized through the transfer of a hydrogen atom or an electron (or both, in sequence) made available by antioxidant polyphenols. The antioxidant activity of a sample is referenced against the scavenging activity of a standard reducing molecule at a known concentration (i.e., Trolox), or as a percentage of radical scavenging, corresponding to the lowering of the absorption peak of the radical species (517 nm) over time [10,11].

The DPPH• procedure involves incubation of the reaction mixture for a set, but variable, time period (from minutes to hours), before reading the decrease in absorbance at 517 nm. However, it has been shown that under some experimental conditions this time duration is not sufficient to reach the steady state. The kinetics of radical scavenging is very fast in the first step with many polyphenols, but the reactive polyphenol (Phen-O•) obtained after HT or ET processes can undergo further reactions, influencing the overall stoichiometry (i.e., the number of radical molecules reduced by a single molecule of reductant). Previous studies have demonstrated that a slight but continuous decrease in absorbance could still be observed up to 24 h of incubation [12,13]. The stoichiometry of the overall reaction is strongly influenced by the fate of radical species formed after reduction of DPPH•. The incubation time can be lowered through sample dilution, but this might complicate the assay procedure, requiring time-consuming trials in order to determine the optimum dilution conditions. The ratio of the effective content of active polyphenols to the actual weight of the extract powder is highly variable among different commercial formulations, so that a standard dilution value cannot be applied.

The purpose of this work is to evaluate the results of DPPH• measurements on oenological tannin solutions prepared at a standard powder weight, and to compare these results with the CV method, considered as a potential alternative to the controversial spectrophotometric method, affected by the variable time needed to run the assay and possible inferences due to its application in coloured samples. The main oenological tannins and their basic monomeric fractions show redox properties that were already elucidated in previous works [7,14,15]. Gallic acid, (+)-catechin and (−)-epicatechin moieties, exhibit low oxidation potentials among polyphenols due to the ortho-diphenol substitution of their aromatic rings. These molecular features give a first oxidation peak close to 400 mV (compared to Ag/AgCl) in a model wine solution [14,16].

It is reasonable to measure the charge passed to 500 mV (integrated peak of the voltammogram), obtaining a parameter comparable to the DPPH• response. This is due to the low oxidation potential of the synthetic DPPH• radical itself, which exhibits a reversible redox couple at about 100 mV more positive than catechol-containing polyphenols; the DPPH• radical can oxidize catechol, pyrogallol and related moieties, but is less effective in reducing benzoic acids, some hydroxycinnamic acids like coumaric acid, or flavonoid A-ring polyphenols. A scan to potentials higher than 500 mV would capture the latter phenolic groups that do not provide significant contribution to the DPPH• radical scavenging mechanism [17].

Previous correlations between DPPH• and electrochemical methods for determination of the antioxidant activity of polyphenols have been attempted so far with satisfactory results [7,18,19,20]. In the present work, commercial tannin powders were dissolved in a medium reproducing wine conditions (hydro-alcoholic buffer solution, pH 3.6) and were assessed using both CV and DPPH• methods. For the spectrophotometric method, different incubation times were evaluated in order to obtain steady-state conditions, and the time-dependent results were correlated with the integrated peak current provided by CV. The potential use of CV as a fast and easy-to-use analytical tool for monitoring the protective action of oenological additives was evaluated, as an alternative to the reliable but time-consuming DPPH• antioxidant assay which is routinely used in the laboratories.

## 2. Results and Discussion

### 2.1. Cyclic Voltammetry

**Figure 2:**
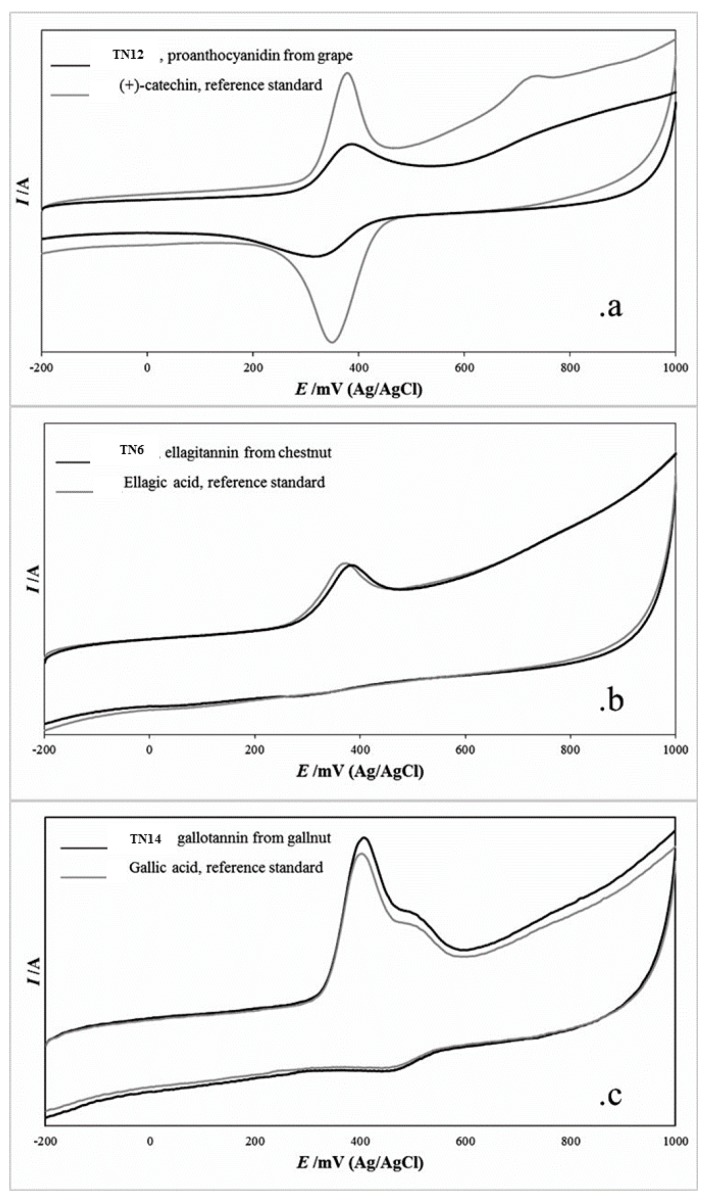
Cyclic voltammograms (background subtracted) for the couples: proanthocyanidin TN12/(+) – catechin; (**a**) ellagitannin TN6/ellagic acid; (**b**) gallotannin TN14/gallic acid; (**c**) in the model wine solution, measured at 100 mV/s above the potential range: −200 mV to 1000 mV, at a 3 mm glassy carbon electrode.

The electrochemical and redox properties of tannins were investigated by analyzing the voltammetry profiles. Voltammograms of representative commercial extracts recorded over a wide range of potentials (−200 mV to 1000 mV) are shown in Figure 2, along with the relevant monomer which is expected to contribute to the polymeric structure, according to the chemical class (Figure 2a: Proanthocyanidin/(+)-catechin; Figure 2b: Ellagitannin/ellagic acid; Figure 2c: Gallotannin/gallic acid). Despite the fact that the whole peak area was used to determine the redox activity, the maximum of electrochemical peaks was considered suitable for a qualitative analysis and to attempt identification based on the botanical sources. The main redox peaks, which are listed in Table 1, are almost overlapping in the potential range up to 500 mV, which is the range of interest for the most active molecular structures, in agreement with electrochemical parameters which were previously reported in the literature for polyphenolic monomers [14,21].

When considering pure condensed tannins (proanthocyanidins), the position of the electrochemical peaks matched with the redox couple found in the (+)-catechin standard. TN2, a tannin extracted from green tea leaves, showed an occasional early oxidation peak at 313 mV with reversible cathodic peak (E_p,c_) at 294 mV; this electrochemical feature was not observed in the other proanthocyanidins of the series, and could be tentatively attributed to the peculiar composition in the galloylated and galloyl/gallate flavanols expected in tea leaves extracts [22,23,24]. The main redox couple confirmed the voltammetry profile expected for condensed tannins-based extracts, with an anodic peak at 419 mV related to a highly reversible process (E_p,a_ − E_p,c_ = 28 mV).

The presence of a prominent, reverse cathodic peak, which is clearly observed in samples TN7 and TN12, which are grape-derived extracts, was related to the occurrence of the redox couple ortho-diphenol/quinone formed during the oxidation process; this can be considered an electrochemically reversible process, despite the peak separation values of the redox couple composed by the anodic and cathodic peak, E_p,a_ – E_p,c_ (ΔE) found in most of the proanthocyanidins investigated were larger than the 29 mV theoretical value expected for a fully reversible system. A value of ΔE = 35 mV was obtained for the (+)-catechin monomeric standard. The presence of highly oxidizable substrates with high reversibility, reflect the peculiar antioxidant capacity of proanthocyanidins, and will be discussed in more detail in Section 2.3.

TN8, is a blended formulation including ellagitannins and proanthocyanidins; the prevalence of (+)-catechin – related oxidation peaks among the overlapping electrochemical features confirm that the proanthocyanidins are prevailing in this formulation.

In the case of ellagitannins and gallotannins sourced from tara, gall, oak wood, chestnut, fruit tree wood, and their blended formulations, the anodic oxidation peaks were located in the range 370 mV–406 mV and related to largely irreversible oxidation processes, as a confirmation of the electrochemical features observed in the ellagic acid and gallic acid monomers. Nevertheless, small amounts of flavanol compounds present in wood extracts and blended formulations (TN3, 5, 14, 18) could contribute to the small return cathodic peak observed.

A further sub-grouping was assigned for the anodic oxidation peaks around 370 mV–386 mV and 399 mV–406 mV. The first grouping was attributed to the gallic and ellagic acid carboxylic moieties (-COOH), which is largely involved in the antioxidant activity of these polyphenolic compounds and their derivatives; the second grouping may be related to the occurrence of esterification and/or coordination to central sugar moieties, which are recurring in hydrolysable extracts as previously disclosed by MALDI-TOF investigations [24,25].

### 2.2. Practical Issues Related to the Use of DPPH• Assay to Determine Antiradical Activity of Tannins

The DPPH• colorimetric assay is based on the ability of reducing molecules to scavenge the 2,2-diphenyl-1-picrylhydrazyl synthetic radical dissolved in an alcoholic solution, thus obtaining its neutral form. The process is monitored through the conversion of the violet-colored radical with the absorbance at 517 nm into a pale-yellow solution, and the antiradical activity was reported at a (%) decrease of such absorbance at different incubation times: 15 min, 30 min, 60 min, 180 min, and 24 h. The overall kinetic curve describing the radical drop up to 24 h gained increasing complexity while increasing the incubation time; nevertheless, all tannins reported the same kinetic trend when considering the decrease in the 517 nm absorbance up to 60 min incubation, highly fitted with zero-order kinetic rates (R^2^ > 0.90). The partial kinetics corresponding to the primary, fast step of DPPH• scavenging by tannins (see Figure 1) are reported in the Table 2.

The 60-min incubation step corresponds to the initial “fast step” rate of DPPH• scavenging, involving the one-electron or one-proton transfer processes and conversion into quinones. While this represents the main reaction involved in the radical scavenging process, it is not exhaustive, since a variable number of by-products are produced during this process (some are shown in Figure 2 for (+)-catechin-based molecules), that could further contribute to the radical scavenging activity. The secondary antiradical reactions occur at a later stage, as an increasing number of functional groups within the molecules are involved; this is the reason for the complex kinetic curves and long-term incubation period needed to reach the steady state by using tannins as scavenger molecules. Due to the complex chemical structure of tannins the 1-h incubation time, which is usually recommended in literature for the DPPH• assay, do not provide a full description if the antiradical activity of these compounds. The radical scavenging clearly continued in the later incubation period until the steady state was reached for all the tannins assayed, regardless of the nature of the extracts.

When considering the overall reaction period and performing periodical comparisons between DPPH• values and the CV response obtained for tannins, we obtained a good correlation, which tended to increase with longer incubation times (data not shown). Best performances were obtained comparing DPPH• and CV results after 24 h incubation time (steady-state), with satisfactory model fitting parameters (R^2^ 0.925; MSE 0.000; RMSE 0.020; standard error 0.065). The DPPH• and CV results obtained at the steady state are listed in Table 3 (Section 2.3).

### 2.3. Antioxidant Activity of Tannins

Combining results obtained by the DPPH• colorimetric assay under steady-state conditions and cyclic voltammetry measurements, information was gained about the redox properties and radical scavenging activity induced by electron and hydrogen-transfer, which are key processes related to the antioxidant capacity of tannins. The anodic peak occurring at around 500 mV in the voltammograms accounted for the oxidation of catechol and pyrogalloyl moieties of polyphenolic compounds. These same moieties react readily with the DPPH• radical itself, a weak oxidizing agent. The ortho -di (catechol) and -tri (pyrogallol) hydroxyl substitutions on flavonoids and benzoic acids increase the antioxidant activity of these compounds, which are similarly captured by the two analytical approaches [14].

Table 3 reports antiradical (%) and redox (mM CE) activities for the extracts selected in this study. Among them, samples TN2 exhibited a strong antioxidant activity, in agreement with the redox current measured under the 500 mV peaks (TN2:DPPH• = 83.4%, CV 500 mV = 0.365 TN14:DPPH• = 74.6%, CV 500 mV = 0.338 mM CE). The sample TN2 is obtained by extracting green tea leaves; despite it belonging to the proanthocyanidin-based tannins group, its reducing properties are improved when compared to the condensed tannins TN5 and TN12, extracted from grapes (TN5: DPPH• = 48.5%, CV 500 mV = 0.221; TN12: DPPH• = 40.9%, CV 500 mV = 0.158 mM CE). It was previously observed [26] that galloylation introduced on the flavanol-based compounds increases their effectiveness as radical scavengers, and this structure is likely to occur in proanthocyanidins derived from green tea leaves and grape seeds [27].

The extract TN7, obtained from grape seeds, showed similarity with TN14, extracted from tara and gallnut rich in tannic acid and gallic acid-based structures; their redox and scavenging activity were comparable under the present experimental conditions (TN7: DPPH• = 70.9%, CV 500 mV = 0.335 mM CE; TN14 DPPH• = 74.6%, CV 500 mV = 0.338 mM CE), possibly due to their similar composition. In both cases the compounds are characterized by a carboxylic function (gallic acid units in the gallotannin and galloyl and gallate substituents in the grape seed proanthocyanidin), with enhanced antiradical effects [28,29].

The ellagitannins assayed and obtained from American oak (TN10) and French oak (TN11), together with samples TN15, TN16, TN17 and TN20 also extracted from oak with generic geographical origin, are considered key tannins to be exploited in the wine industry, resembling the long-term storage of fine wines in barrels. Nevertheless, TN10 and TN11 showed the lowest radical scavenging (DPPH• = 30.2% and 38.2% of radical scavenged, respectively) and redox values (CV 500 mV = 0.115 and 0.133 mM CE, respectively) of the ellagitannin series (TN15, TN16, TN17 and TN20 average DPPH• = 51.8 ± 1.65%; average CV 500 mV = 0.221 ± 0.0158 mM CE). The occasional drop in TN10 and TN11 was ascribed to a lower effectiveness of the extraction process and reduced content of bioactive compounds more than to the intrinsic composition of these extracts, this hypothesis requires further confirmation by analyzing the effective polyphenolic content of the extract.

Some blended formulations are included in the sample set and they showed variability in terms of fractions of different tannins added, which resulted in variable scavenging activities and electrochemical redox values observed. The interpretation of results and definition of structure-activity would require more complex analytical techniques for the definition of the compositional profiling of the blends, and the relative contribution of active fraction involved in the antioxidant mechanisms.

The use of tannins in oenology is gaining increasing attention due to their unique technological properties, sensory impact, and nutraceutical value [5,30,31,32]. Since they can be both sourced from grapes and added as exogenous products during winemaking, a proper understanding of the compositional properties and bioactivity of commercial formulations is a challenging opportunity for winemakers. The antioxidant activity of tannins is considered an opportunity to lower the use of potentially harmful additives, i.e., SO_2_, and to preserve the shelf-life of wines without altering their sensory perceptions.

## 3. Conclusion

Along with dedicated analytical methods which are exploited in wine research, there is a need for rapid and reliable analytical tools, with simplified instrumentation and data output to obtain technological information and support decisions along the supply chain. This also includes fast methods which provide information about the correlation between the dosage of the commercial products and related bioactivity.

In this work, spectrophotometric (DPPH•) and electrochemical (CV) methods were compared according to their ability to disclose the antioxidant activity of botanical extracts used in oenology for protective purposes. The DPPH• measurements confirmed previous observation about potential limits of this colorimetric assay. The main issue was the long time required to obtain a realistic representation of the antioxidant capacity, which is possibly due to the complex chemical structures involved in the antioxidant reactions, together with the number of by-products and side-reactions contributing to the overall antiradical effect. The CV measures was shown to be well correlated with the DPPH• values obtained at the steady state. Moreover, compositional considerations on the extracts can be derived from the voltammetry profiles.

CV is a fast analytical approach for the study of the antioxidant capacity of tannin compounds, giving reliable and reproducible results. It does not require special reagent mixtures and long incubation times, and the only reactant required is a supporting electrolyte with a known pH value, where the sample is dissolved to a suitable concentration. Moreover, only thermodynamic properties of the molecules are involved in the electrochemical measurements, without being affected by further properties of the sample, such as color and turbidity, which strongly influence the spectrophotometric assays. Given these considerations, CV is well suited for quality control, providing a fast and reliable method to monitor the redox activity of tannin additives for use in the wine supply chain, to modulate dosages and to improve the protection of wines and musts against oxidation.

## 4. Materials and Methods

### 4.1. Tannins

Twenty tannins commercially available as lyophilized, food-grade powders for oenological use were selected for this case study, accounting for the variability of the botanical sources. General information on their composition is reported in Table 4, as provided by the product suppliers (Enologica Vason, Verona, Italy; HTS Enologia, Marsala, Italy; Laffort, Bordeaux Cedex, FR; AEB Group, Brescia, Italy). Stock solutions of dry tannins were prepared at 1 g/L in a model wine solution, and further diluted for analytical purposes. The model wine solution was made up of 12% *v*/*v* ethanol (> 99%) in distilled water, with the addition of L-tartaric acid 0.033 M and NaOH to reach pH 3.6. The model wine buffer was prepared using Merck HPLC- grade reagents without further purification (Merck, Darmstadt, Germany). Stock solutions of monomeric standards: (+)-catechin, gallic acid, ellagic acid, were prepared under the same conditions for comparative purposes.

### 4.2. Chemicals and Reagents

The 2,2-diphenyl-1-picrylhydrazyl (DPPH•) free radical (95%) and pure methanol HPLC-grade (99.8%) used for the spectrophotometric assay, together with the (+)-catechin monohydrate analytical standard (98%) used to calibrate the CV measurements, gallic acid and ellagic acid standards, were purchased from Sigma-Aldrich (Sigma-Aldrich, Saint Louis, MO, United States).

### 4.3. DPPH• Assay

The DPPH• assay was performed according to the originally proposed method [11] modified by Villaño et al. [10]. Briefly, 100 μL of tannin solutions containing 58 mg/L powders were added to 2.9 mL of 200 μM DPPH• (molar mass: 394.32 g/mol) in methanol. The resulting solutions were vigorously shaken and hermetically sealed to prevent solvent evaporation, incubated in the dark at room temperature and sampled after 15 min, 30 min, 60 min, 180 min incubation, respectively, until reaching the steady state of reaction (1440 min, 24 h). The absorbance was measured at 517 nm in 10 mm plastic cuvettes against pure methanol, using a Shimadzu UV mini 1240 spectrophotometer (Kyoto, Japan). The results were expressed as percentage of inhibition [30], using the following formula:

%inhibition = [(Ab − As)/Ab] × 100

where: Ab = absorbance of the reagent blank, and As = absorbance of the sample. Each experiment was run in triplicate and results are provided as average values.

Calculation of the zero-order kinetics related to the fast step of the DPPH• radical scavenging reaction by tannins were performed using the Shimadzu UVProbe software connected to the Shimadzu UV mini 1240 spectrophotometer in the kinetics (time-course) measurement mode (Shimadzu, Kyoto, Japan). The measurements were performed at the wavelength of 517 nm, starting from the mixing of tannins and DPPH• solutions (time zero) and setting a total of 30 subsequent readings separated by time steps of 120 s (3600 s total time). The resulting absorbance values (a.u.) compared to the elapsed time (s) were used to calculate the kinetic rates; results were expressed as abs_517nm_ s^−1^ and are reported in Table 2 (Section 2.2).

### 4.4. Cyclic Voltammetry

The same dilution used in the spectrophotometric experiment was found suitable for CV experiments, performed according to the method previously described [14]. The following equipment was used for CV analysis: A Bioanalytical Systems (BAS) 100A electrochemical analyzer, a BAS C2 electrochemical cell, a 3 mm glassy carbon disc electrode (working electrode, BAS M-2012), a platinum counter electrode and an Ag/AgCl reference electrode (+207 mV compared to SHE). The glassy carbon electrode was polished with 3 μm alumina powder (PK-4 polishing kit), then rinsed with ultrapure 18 MΩ water, to avoid surface passivation and poisoning. The scan range was taken from −200 mV to 500 mV, in order to record the first anodic peak, related to reactive polyphenols. The voltammograms were recorded at a scan rate of 100 mV/s and a sensitivity of 1 μA. A blank run was recorded daily in the model wine solution and was used to subtract the background current. Each experiment was run in triplicate and the average values of the increase in anodic current with potential, μA × mV (current integrated) were expressed in mM (+)-Catechin Equivalents (CE), using a calibration curve of the standard over the range of 0.0078 mM to 1.0 mM.

### 4.5. Data Processing

Microsoft Excel was used for data entry, whereas derivatives of the cyclic voltammograms were calculated using OriginPro 8 (Origin Lab Corp., Northampton, MA, USA). All analyses were performed in triplicate and results were provided as average values; statistical analyses were performed with the XLSTAT (Addinsoft 2018, Paris, France) software.

## Figures and Tables

**Table 1:** Anodic (E_p,a_) and cathodic (E_p,c_) peak potentials obtained by scanning tannin solutions in the range of −200 mV to 500 mV. The redox couples obtained by reversible processes are labelled in italics. A detailed description of tannin samples (TNs) according to the information by suppliers is provided in Table 4 of the ‘Materials and Methods’ section.

Samples	E (mV versus Ag/AgCl)			
	E_p,a_		E_p,c_	
(+)-Catechin, std	-_	*389*	-_	*354*
Ellagic acid, std	-_	367	-_	-_
Gallic acid, std	-_	*391*	-_	-_
TN1	-_	402	-_	-_
TN2	*313*	*419*	*294*	*391*
TN3	290	*383*	-_	*329*
TN4	-_	406	-_	_
TN5	308	406	-_	*337*
TN6	-_	384	-_	_
TN7	-_	*399*	-_	*338*
TN8	290	*399*	-_	*332*
TN9	-_	404	-_	-_
TN10	-_	386	-_	-_
TN11	-_	380	-_	-_
TN12	-_	*399*	-_	*321*
TN13	-_	401	-_	-_
TN14	-_	*413*	-_	*395*
TN15	-_	379	-_	-_
TN16	-_	370	-_	-_
TN17	-_	382	-_	-_
TN18	-_	*384*	-_	*328*
TN19	-_	396	-_	-_
TN20	-_	404	-_	-_

**Table 2:** Zero-order kinetic rates of DPPH• radical scavenging by tannins over a 60-min incubation period.

Samples	DPPH• Scavenging Kinetic Rates [0–60 min Incubation]
Code	% decrease Abs_517 nm_/min
TN1	0.139
TN2	0.085
TN3	3.059
TN4	0.106
TN5	0.091
TN6	0.064
TN7	0.167
TN8	0.115
TN9	0.130
TN10	0.060
TN11	0.070
TN12	0.133
TN13	0.129
TN14	0.196
TN15	0.102
TN16	0.074
TN17	0.097
TN18	0.118
TN19	0.087
TN20	0.076

**Table 3:** DPPH• radical scavenging values (%) at the steady-state (24 h incubation time), and charge passed to 500 mV (converted to mM (+)-catechin equivalent) for the tannins investigated.

Samples	DPPH• Radical Scavenging (%) at the Steady State (24 h)	CV −200 mV to 500 mV
Code	% decrease Abs_517 nm_	mM CE
TN1	49.8 ± 0.1	0.225 ± 0.001
TN2	83.4 ± 0.8	0.365 ± 0.008
TN3	47.0 ± 0.3	0.187 ± 0.006
TN4	51.5 ± 1.2	0.258 ± 0.008
TN5	48.5 ± 0.1	0.221 ± 0.008
TN6	69.3 ± 0.4	0.349 ± 0.002
TN7	70.9 ± 1.1	0.335 ± 0.006
TN8	59.1 ± 0.1	0.311 ± 0.002
TN9	63.8 ± 0.6	0.313 ± 0.005
TN10	30.2 ± 1.2	0.115 ± 0.004
TN11	38.2 ± 3.2	0.133 ± 0.007
TN12	40.9 ± 1.2	0.158 ± 0.008
TN13	55.0 ± 0.6	0.234 ± 0.007
TN14	74.6 ± 0.1	0.338 ± 0.006
TN15	53.9 ± 0.2	0.232 ± 0.011
TN16	52.0 ± 0.5	0.233 ± 0.004
TN17	50.0 ± 0.1	0.199 ± 0.003
TN18	62.8 ± 0.2	0.282 ± 0.005
TN19	57.0 ± 0.1	0.237 ± 0.003
TN20	51.1 ± 0.1	0.219 ± 0.003

**Table 4:** Commercial oenological tannins selected for this work with the generic compositional information provided by suppliers. The classification as “Blend “refers to the mixture of tannins from different botanical sources as described in the commercial label.

Code	Chemical Classification	Botanical Origin
TN1	Ellagitannin	White fruits tree wood
TN 2	Proanthocyanidin	Green tea
TN 3	Proanthocyanidin	Unknown
TN 4	Blend, not specified	Unknown
TN 5	Proanthocyanidin	Grape
TN 6	Ellagitannin	Chestnut heartwood
TN 7	Proanthocyanidin	Grape seed
TN 8	Blend: proanthocyanidin/ellagitannins	Unknown
TN 9	Blend: proanthocyanidin/ ellagitannin/gallotannin	Limousin French oak, tara, gall, green tea
TN 10	Ellagitannin	American oak
TN 11	Ellagitannin	French oak (Allier)
TN 12	Proanthocyanidin	Grape
TN 13	Blend: proanthocyanidin/ ellagitannin/gallotannin	Limousin French oak, gall, grape
TN 14	Not specified	Unknown
TN 15	Ellagitannin	French oak (Limousin)
TN 16	Ellagitannin	Selected *Quercus* woods
TN 17	Ellagitannin	French oak
TN 18	Ellagitannin	Red fruit tree wood
TN 19	Blend: proanthocyanidin/ ellagitannin/gallotannin	Tara, gall, green tea, ellagitannins from oak
TN 20	Ellagitannin	French oak (Allier)
